# Modulation of the Cardiovascular Effects of Polycyclic Aromatic Hydrocarbons: Physical Exercise as a Protective Strategy

**DOI:** 10.3390/toxics11100844

**Published:** 2023-10-08

**Authors:** Gabriel A. Rojas, Nicolás Saavedra, Cristian Morales, Kathleen Saavedra, Fernando Lanas, Luis A. Salazar

**Affiliations:** 1 Center of Molecular Biology & Pharmacogenetics, Department of Basic Sciences, Scientific and Technological Bioresource Nucleus (BIOREN), Universidad de La Frontera, Temuco 4811230, Chile or gabriel.rojas5@cloud.uautonoma.cl (G.A.R.); nicolas.saavedra@ufrontera.cl (N.S.); c.morales15@ufromail.cl (C.M.); kathleen.saavedra@ufrontera.cl (K.S.); 2 PhD Program in Applied Cellular and Molecular Biology, Universidad de La Frontera, Temuco 4811230, Chile; 3 Escuela Kinesiología, Facultad de Ciencias de la Salud, Universidad Autónoma de Chile, Talca 3460000, Chile; 4 Tecnología Médica, Facultad de Salud, Universidad Santo Tomás, Temuco 4811230, Chile; 5 Department of Internal Medicine, Faculty of Medicine, Universidad de La Frontera, Temuco 4811230, Chile; fernando.lanas@ufrontera.cl

**Keywords:** polycyclic aromatic hydrocarbons, cardiovascular risk, aerobic exercise

## Abstract

Exposure to polycyclic aromatic hydrocarbons (PAHs) present in air pollution increases cardiovascular risk. On the contrary, physical exercise is a widely used therapeutic approach to mitigate cardiovascular risk, but its efficacy in an environment of air pollution, particularly with PAHs, remains unclear. This study investigates the effects of exercise on inflammation, endothelial dysfunction, and REDOX imbalance due to PAH exposure using a mouse model. Twenty male BALB/c mice were subjected to a mixture of PAHs (phenanthrene, fluoranthene, pyrene) in conjunction with aerobic exercise. The investigation evaluated serum levels of inflammatory cytokines, gene expression linked to inflammatory markers, endothelial dysfunction, and REDOX imbalance in aortic tissues. Furthermore, the study evaluated the expression of the ICAM-1 and VCAM-1 proteins. Exercise led to notable changes in serum inflammatory cytokines, as well as the modulation of genes associated with endothelial dysfunction and REDOX imbalance in aortic tissue. In turn, exercise produced a modulation in the protein expression of ICAM-1 and VCAM-1. The findings implicate the potential of exercise to counter PAH-induced damage, as demonstrated by changes in markers. In conclusion, exercise could mitigate the adverse effects related to exposure to PAHs present in air pollution, as evidenced by changes in inflammatory markers, endothelial dysfunction, and REDOX imbalance.

## 1. Introduction

Air pollution, both environmental pollution and household air pollution, is a major global problem that generates a serious health risk [1]. According to the World Health Organization (WHO), worldwide environmental and domestic air pollution affects 90% of the world’s population, causing at least seven million preventable deaths each year [2]. Air pollution is generally described in terms of the pollutants present in it: particulate matter (PM), ozone (O_3_), sulfur dioxide (SO_2_), oxides of nitrogen (NOx), carbon monoxide (CO), benzene, and lead (Pb); of these, PM affects more people than any other pollutant [3]. Particulate matter is classified according to the diameter of its particles: PM_10_ has a diameter less than 10 µm, PM_2.5_ less than 2.5 µm, and PM_0.1_ less than 0.1 µm. PM_2.5_ is a complex mixture of several constituents with different physicochemical properties and toxicity, and the total composition of particles varies according to the source of emission and the season of the year [4]. PM_2.5_ corresponds to the most studied component of air pollution and the one for which world regulations place the greatest emphasis on its control due to documented deleterious effects, classifying it as a group 1 carcinogen [5,6]. At the same time, exposure to polycyclic aromatic hydrocarbons (PAHs) found in different types of smoke could be linked to certain diseases. Mixtures containing PAHs can potentially lead to various adverse effects on the human body, including mutagenic effects and increased susceptibility to cancer development. As a result, PM and PAHs are among the most harmful air pollutants in terms of health [7,8,9].

PAHs are produced by incomplete combustion of organic materials such as coal and fossil fuels, wood burning, cigarette smoking, and various industrial activities. It has been estimated that the main sources of total global air emission of PAHs are residential/commercial biomass burning (60.5%), open-field biomass burning (agricultural residue burning, deforestation, forest fires (13.6%)), and motor oil consumption by vehicles (12.8%) [10]. The concentration of these compounds depends on several factors, such as season of year, geographical location, and population. It was determined in Temuco, Chile, that the predominant individual PAHs were phenanthrene (35–45%), fluoranthene (11–15%), and pyrene (9–12%), typical characteristics of biomass combustion, especially the combustion of wood for heating or cooking [11]. 

Three main mechanisms by which air pollution affects the cardiovascular system have been proposed: (1) secretion of inflammatory mediators and oxidative stress in the cardiovascular system; (2) imbalance of the autoimmune nervous system; and (3) direct penetration of particles and components into the cardiovascular system [12]. Exposure to PAHs in the workplace and in the environment may lead to the development of cardiovascular disease (CVD), such as coronary artery disease, peripheral arterial disease, stroke, and myocardial infarction [13]. Physical exercise, on the other hand, plays an essential role in the management of cardiovascular risk factors such as hypertension, endothelial dysfunction, lipid toxicity, obesity, and diabetes. Therefore, body composition, blood pressure, lipid profiles, and glycemia control are significant in patients at risk of CVD when exercising, and patients with coronary disease and heart failure observe great health benefits [14,15,16].

Despite positive evidence in favor of physical exercise, the literature on the habitual practice of physical exercise in the context of air pollution is controversial. On the one hand, participating in physical activity requires an intake of oxygen, and the oxygen demand increases as the intensity of the exercise increases. Mouth breathing becomes more common with exercise, bypassing nasal filtration, increasing the amount of inhaled pollution and the degree to which it moves into the respiratory system, and creating high risk, especially for subjects diagnosed with morbidities [17]. However, there is evidence that in the long term, the beneficial effects of physical exercise on cardiovascular health are more relevant than the harmful effects caused by air pollution [18,19]. Accordingly, in animal models, a decrease in lung inflammation has been found, mediated by a better antioxidant response in mice subjected to high-intensity aerobic exercise in conjunction with exposure to pollutants derived from diesel exhaust particles (DEPs), PM_2.5_, and PM_10_ [20,21,22].

Almost all studies related to exercise and air pollution have used PM_10_, PM_2.5_, and DEP as pollution measures; however, there is little evidence related to response to exercise under exposure to PAHs. Therefore, the objective of this investigation was to determine the effect of aerobic physical exercise on cardiovascular disease markers in animals exposed to PAHs derived from air pollution.

## 2. Materials and Methods

### 2.1. Animals

Forty-eight male BALB/c mice were randomly assigned to four equal groups of twelve animals each, including a control group (no exposure or control); an aerobic exercise group (exercise); a group exposed to 50 µg of a PAH mixture (PAHs); and an exercise + exposure to 50 µg of a PAH mixture group (exercise + PAHs). The PAH mixture consisted of 55% phenanthrene (Sigma-Aldrich, St. Louis, USA), 25% fluoranthene (Sigma-Aldrich, St. Louis, MO, USA), and 20% pyrene (Sigma-Aldrich, St. Louis, MO, USA). USA), proportional to the most representative distribution of the PAHs previously described [11]. Dimethylsulfide (DMSO, Sigma-Aldrich, St. Louis, MO, USA) was used as a solvent. The animals were kept in the Bioterium of the University of La Frontera and fed standard food with ad libitum water access.

### 2.2. Nasal Instillation

Animals were acclimatized for two weeks according to the previously described nose instillation protocol [23]. All groups were administered an inhaled nasal volume of 10 µL using a micropipette. Nasal instillation led to reflexes of apnea and deep inspiration. Therefore, the control group and the aerobic exercise group were instilled with a vehicle solution, and the groups exposed to PAHs and to exercise + PAHs were instilled with the mixture of PAHs.

### 2.3. Intervention Protocol

The intervention protocol consisted of the instillation of a vehicle or a mixture of PAHs, depending on the group, in addition to exercise, depending on the group, 5 days a week for 5 weeks. For groups that did not exercise (control and exposure), 10 min after nasal instillation, mice were placed on a treadmill for the same time as groups that exercised, but with speed 0. Each week, the weight of the animal was recorded. To evaluate the general situation of the animals, the Morton and Griffiths Animal Monitoring Protocol [24] was applied once a week. The Scientific Committee on Ethics of the University of La Frontera (No. 105_18) approved the experimental protocol.

### 2.4. Exercise Training

Before starting treadmill training, the animals were acclimatized to the animal-adapted treadmill (treadmill, Bianchi BFT 2525) with a 6-lane acrylic box for 2 weeks, 5 times a week, starting with 5 min at speed 0 up to 15 min at 20 m/min by the end of the acclimatization period. Subsequently, a maximum exercise capacity test was performed that evaluated maximum distance, length, and speed, starting at 16.7 m/min, increasing speed every 2.5 min by 3.33 m/min until fatigue, defined as the animal’s inability to reach the end of the lane after being stimulated with five mechanical stimuli (soft brush) administered in 1 min [25].

This allowed us to determine the intensity of the physical training that was set at a moderate intensity of exercise corresponding to 70% of the maximum speed. The treadmill training was performed 5 times a week, 40 min per session, for 5 weeks. A final maximum exercise capacity test was performed 48 h before euthanasia of the animals.

### 2.5. Sampling Extraction

Euthanasia was performed with a mixture of ketamine/xylazine with a lethal intraperitoneal dose of 200 mg/kg of ketamine-16 mg/kg of xylazine. A whole blood sample was collected by cardiac puncture and centrifuged at 2000 rpm for 15 min. Once serum was separated from the blood clot, the sample was stored at −80 °C for subsequent analysis. The thoracic artery was removed and stored at −80 °C in a sterile tube with a 1 mL RNAlater^TM^ stabilizer solution (Ambion Inc., Austin, TX, USA).

### 2.6. Serum cytokines 

Serum cytokine levels were analyzed using the 6-Plex Kit for the Bio-Plex Pro Mouse Cytokine Th17 Panel A (BioRad, Hercules, CA, USA), following the manufacturer’s instructions, with the MAGPIX^®^ system (Luminex, Austin, TX, USA). This kit includes the analysis of IL-6, IL-1β, TNF-α, INF-gamma, IL-17A, and IL-10. The samples were run in duplicate, and an 8-point standard curve was included in duplicate, in addition to two wells as negative controls. The raw values were obtained with the xPONENT 4.2^®^ software (Luminex, Austin, TX, USA), but for the final analysis, the values obtained for each cytokine were adjusted by the weight of each animal.

### 2.7. RT-qPCR

Gene expression was analyzed by reverse transcription followed by real-time quantitative polymerase chain reaction (RT-qPCR). Specific primers for ICAM-1, VCAM-1, E-selectin, endothelial nitric oxide synthase (eNOS), nuclear factor erythroid-derived 2-like 2 (Nrf2), aryl hydrocarbon receptor (Ahr), transcription factor p65 (RelA), IL-6, and TNF-α, along with reference genes for ribosomal protein L32 (RPL32) and beta-2 microglobulin (B2m), were designed for this study (Table 1). Total RNA samples were obtained from frozen aortic tissue using TRIzol^®^ reagent (Invitrogen, Waltham, MA, USA) following the manufacturer’s recommendations. Tissue was lysed using 2 mL tubes prefilled with ceramic beads (MP biomedical, Solon, OH, USA) in a benchtop BeadBug^TM^ homogenizer (Benchmark Scientific, Sayreville, NJ, USA) for 60 s at 3500 rpm. Subsequently, the purity of the samples was evaluated by spectrophotometry (NanoQuant Infinite^®^ 200 PRO, Tecan^®^, Männedorf, Switzerland) and fluorometry (Quantus^TM^ Fluorometer, Promega, Madison, WI, USA) of the amount of extracted RNA, considering a ratio between 1.8–2.0 as acceptable. Total RNA samples were diluted to ensure a final concentration of 30 ng/µL. cDNA was synthesized by reverse transcription using the High-Capacity cDNA Reverse Transcription Kit (Applied Biosystems, Foster City, CA, USA). qPCR was performed to quantify the expression of each of the genes of interest and reference genes using the Fast SYBR^®^ Green Master Mix Kit (Applied Biosystems, Foster City, CA, USA) following the manufacturer’s protocols. For qPCR analysis, LinRegPCR^®^ software (version 2013.1; http://LinRegPCR.nl, accessed on 20 December 2022) was used [26].

### 2.8. Western Blotting 

Total protein extraction from aortic tissue was performed using the TRIzol^®^ reagent protocol. Proteins were quantified by the BCA method (Thermo Scientific^TM^, Rockford, IL, USA) and taken at a final concentration of 40 µg for Western blotting assays. Briefly, the protocol used was as follows: 4× Laemmli sample buffer (Bio-Rad, Hercules, CA, USA) was prepared by adding β-mercaptoethanol in a 1:9 ratio (Bio-Rad). To this, the protein extract was incorporated, diluted at a ratio of 3:1. The samples were denatured by increasing the temperature to 95 °C for 5 min in a thermocycler, followed by incorporating the samples into the 4–20% Mini-PROTEAN^®^ TGXTM electrophoresis gel (Bio-Rad). Then, electrophoresis was applied vertically at 100 volts for 15 min and then at 200 volts for 30 min. The proteins were then transferred to a PVDF immunoblotting membrane (Bio-Rad) for 1.5 h with an amperage of 350 mAmp.

Subsequently, the membrane was blocked with 5% NFDM/TBS-Tween for 1 h, then washed and incubated with the primary antibodies at 4 °C overnight according to the manufacturer’s instructions: VCAM-1 (1:1000, 5% BSA, 1× TBS, 0.1% Tween^®^20, Cell Signaling 32653, Danvers, MA, USA), ICAM-1 (1:1000, 5% NFDM, 1× TBS, 0.1% Tween^®^20; Abcam, ab179707), and β-actin loading control (1: 20,000, 5% BSA, 1× TBS, 0.1% Tween^®^20; Abcam ab49900, Cambridge, MA, USA). The membranes were washed and incubated with HRP-conjugated secondary antibody (1:3000, 5% NFDM 1× TBS, 0.1% Tween^®^20, goat anti-rabbit IgG; Cell Signaling 7074, Danvers, MA, USA) for 1 h at room temperature under shaking, except for β-actin, for which an HRP-conjugated primary antibody was used. Finally, using the developing reagent from the West Femto Maximum Sensitivity Substrate Kit (Thermo Scientific^TM^, Rockford, IL, USA), antigen–antibody binding bands were detected with the GBOX Chemi XRQ chemiluminescence kit (SYNGENE, Frederick, MD, USA). Densitometric band analysis was performed using ImageJ 1.51j8 software (https://imagej.nih.gov/ij/index.html, accessed on 20 December 2022, National Institutes of Health, Bethesda, MD, USA).

### 2.9. Statistics

Data analysis was performed with Prism 8.0.2 software (GraphPad, San Diego, CA, USA). Results are presented as mean ± standard error of the mean (SEM). To assess the data distribution, the Shapiro–Wilk normality test was performed. For two-group comparisons, the paired *t*-test or the nonparametric Wilcoxon test was employed based on data normality. Comparisons involving more than two groups utilized Tukey’s multiple comparison test or Welch’s multiple comparison test, coupled with Tukey’s or Dunnett’s multiple comparison tests. In cases where data distribution assumptions were not met, analogous nonparametric approach was used: Kruskal–Wallis analysis with Dunn’s multiple comparison test. For analyses involving two variables, a two-way analysis of variance (ANOVA) was employed for group comparisons. Statistical significance was established at a *p*-value of 0.05.

## 3. Results

### 3.1. Animals

**Figure 1:**
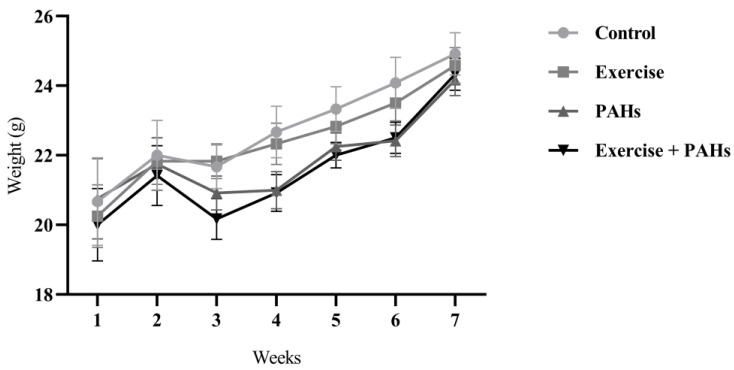
Evolution of weight per week by group (*p* = 0.560). Two-way ANOVA (n = 12 per group).

The initial weights of the animal groups did not show significant differences (*p* = 0.909). The mean weights were as follows: control group 19.58 ± 0.93 g, exercise group 19.00 ± 0.56 g, PAHs group 19.33 ± 0.74 g, and exercise + PAHs group 18.75 ± 0.70 g. Weekly weight measurements were taken, showing a non-significant difference between groups (*p* = 0.560). However, each group demonstrated a statistically significant increase in weight (*p* < 0.0001) throughout the course of the protocol (Figure 1).

### 3.2. Training Effects

**Figure 2:**
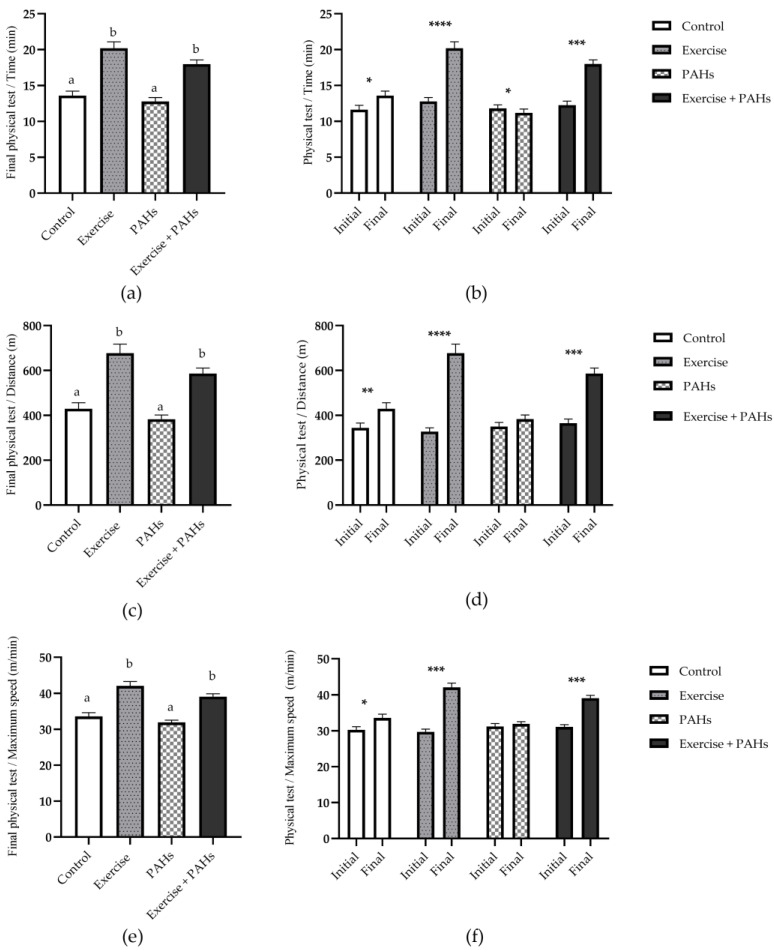
Physical capacity test after the intervention protocol. (**a**) Comparison of time between groups (*p* < 0.0001) (control vs. exercise *p* < 0.0001; control vs. exercise + PAHs *p* = 0.0002; exercise vs. PAHs *p* < 0.0001; PAHs vs. exercise + PAHs *p* < 0.0001) ^A^. (**b**) Intragroup comparison of test time (control *p* = 0.022; exercise *p* < 0.0001; PAHs *p* = 0.048; exercise + PAHs *p* = 0.0001) ^P-T^. (**c**) Comparison of distance between groups (*p* < 0.0001) (control vs. exercise *p* < 0.0001; control vs. exercise + PAHs *p* = 0.0017; exercise vs. PAHs *p* < 0.0001; PAHs vs. exercise + PAHs *p* < 0.0001) ^A^. (**d**) Intragroup comparison of distance (control *p* = 0.005; exercise *p* < 0.0001; PAHs *p* = 0.058; exercise + PAHs *p* = 0.0001) ^P-T^. (**e**) Comparison of maximal speed between groups (*p* < 0.0001) (control vs. exercise *p* = 0.0006; control vs. exercise + PAHs *p* = 0.032; exercise vs. PAHs *p* < 0.0001; PAHs vs. exercise + PAHs *p* = 0.002) ^K-W^. (**f**) Intragroup comparison of maximal speed (control *p* = 0.031; exercise *p* = 0.001; PAHs *p* = 0.594; exercise + PAHs *p* = 0.001) ^W^. Data are presented as mean ± SEM. Different letters show statistically significant differences. * *p* < 0.05; ** *p* < 0.01; *** *p* < 0.001; **** *p* < 0.0001. ^A^ Ordinary one-way ANOVA with Tukey’s multiple comparisons test. ^K-W^ Kruskal–Wallis with Dunn’s multiple comparison analysis. ^P-T^ Paired *t*-test. ^W^ Wilcoxon test. (n = 12 per group).

The initial physical capacity test did not show statistically significant differences in distance (*p* = 0.570), time (*p* = 0.590), or maximum speed (*p* = 0.484). Referring to the comparison between the groups in the final physical capacity test, it showed differences for all variables (*p* < 0.0001). Regarding the analysis before and after the intervention of physical capacity, it showed differences in test time in all groups (control *p* = 0.022; exercise *p* < 0.0001; PAHs *p* = 0.048; exercise + PAHS *p* = 0.0001), distance in all groups except PAHs (control *p* = 0.005; exercise *p* < 0.0001; PAHs *p* = 0.058; exercise + PAHS *p* = 0.0001), and speed in all groups except PAHs (control *p* = 0.031; exercise *p* = 0.001; PAHs *p* = 0.594; exercise + PAHS *p* = 0.001) (Figure 2).

### 3.3. Serum Cytokines

**Figure 3:**
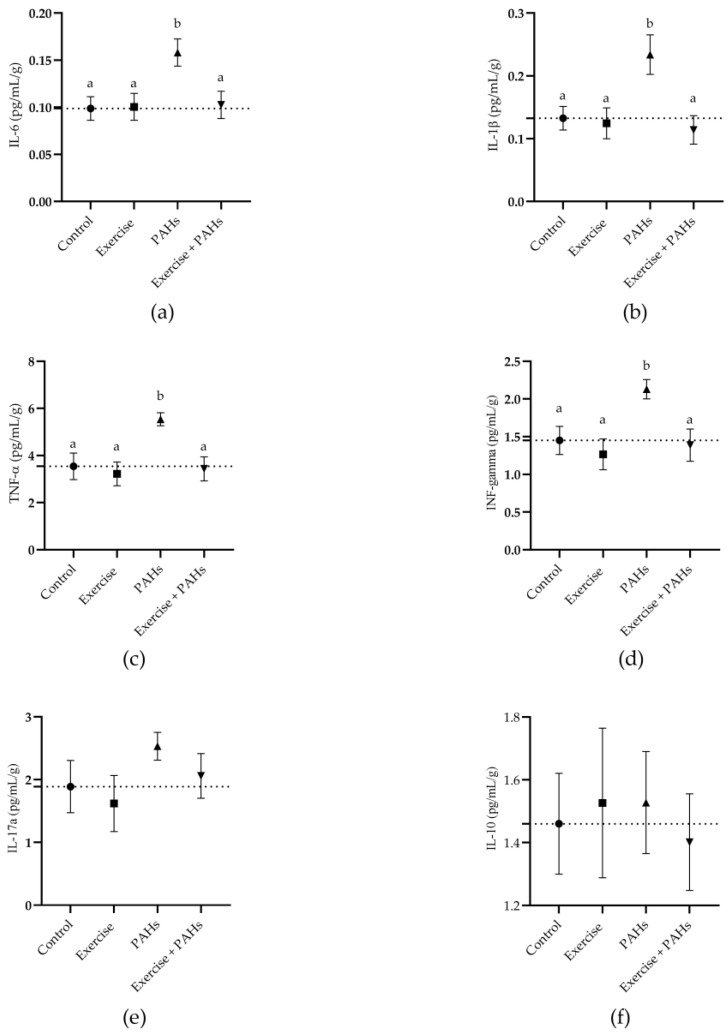
Comparison of weight-adjusted serum inflammatory cytokine quantification between groups. (**a**) IL-6 *p* = 0.017 (control vs. exercise *p* = 0.999; control vs. PAHs *p* = 0.022; control vs. exercise + PAHs *p* = 0.997; exercise vs. PAHs *p* = 0.033; exercise vs. exercise + PAHs *p* = 0.999; PAHs vs. exercise + PAHs *p* = 0.035). (**b**) IL-1β *p* = 0.005 (control vs. exercise *p* = 0.996; control vs. PAHs *p* = 0.029; control vs. exercise + PAHs *p* = 0.951; exercise vs. PAHs *p* = 0.020; exercise vs. exercise + PAHs *p* = 0.991; PAHs vs. exercise + PAHs *p* = 0.009). (**c**) TNF-α *p* = 0.005 (control vs. exercise *p* = 0.968; control vs. PAHs *p* = 0.036; control vs. exercise + PAHs *p* = 0.999; exercise vs. PAHs *p* = 0.009; exercise vs. exercise + PAHs *p* = 0.988; PAHs vs. exercise + PAHs *p* = 0.016). (**d**) IFN-gamma *p* = 0.006 (control vs. exercise *p* = 0.892; control vs. PAHs *p* = 0.045; control vs. exercise + PAHs *p* = 0.995; exercise vs. PAHs *p* = 0.009; exercise vs. exercise + PAHs *p* = 0.968; PAHs vs. exercise + PAHs *p* = 0.031). (**e**) IL-17A *p* = 0.396. (**f**) IL-10 *p* = 0.954. Data are presented as mean ± SEM. The dashed line represents the mean value of the control group. Different letters show statistically significant differences. Ordinary one-way ANOVA with Tukey’s multiple comparison test. (n = 10 per group).

We found significant differences between the intervention and control groups regarding IL-6 (*p* = 0.017), IL-1β (*p* = 0.005), TNF-α (*p* = 0.006), and INF-gamma (*p* = 0.006). On the contrary, IL-17A (*p* = 0.396) and IL-10 (*p* = 0.954) did not have differences between the groups (Figure 3). 

### 3.4. Gene Expression

**Figure 4:**
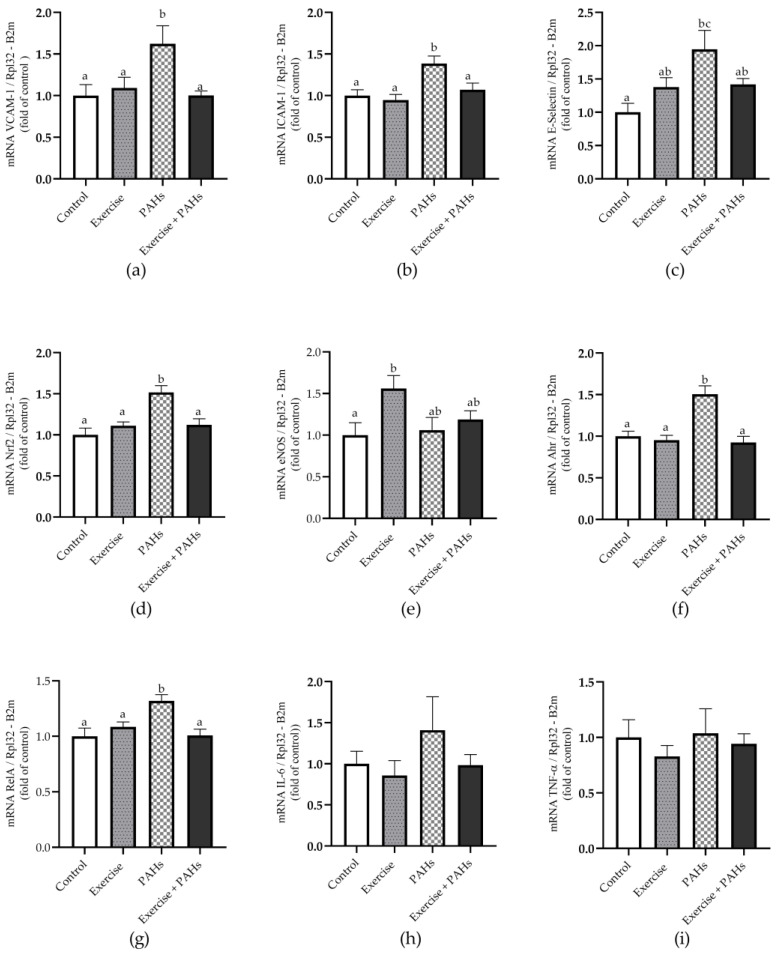
Relative gene expression in aortic tissue of markers of endothelial dysfunction, REDOX imbalance, and inflammation. (**a**) VCAM-1 *p* = 0.005 (control vs. exercise *p* = n.s.; control vs. PAHs *p* = 0.008); control vs. exercise + PAHs *p* = n.s.; exercise vs. PAHs *p* = 0.044; exercise vs. exercise + PAHs *p* = ns; PAHs vs. exercise + PAHs *p* = 0.019) ^K-W^. (**b**) ICAM-1 *p* = 0.001 (control vs. exercise *p* = n.s.; control vs. PAHs *p* = 0.007; control vs. exercise + PAHs *p* = n.s.; exercise vs. PAHs *p* = 0.002; exercise vs. exercise + PAHs *p* = n.s.; PAHs vs. exercise + PAHs *p* = 0.033) ^A^. (**c**) E-selectin *p* = 0.029 (control vs. exercise *p* = n.s.; control vs. PAHs *p* = 0.045; control vs. exercise + PAHs *p* = n.s.; exercise vs. PAHs *p* = n.s.; exercise vs. exercise + PAHs *p* = n.s.; PAHs vs. exercise + PAHs *p* = n.s.) ^W-A^. (**d**) Nrf2 p < 0.0001 (control vs. exercise *p* = n.s.; control vs. PAHs *p* = 0.0001; control vs. exercise + PAHs *p* = n.s.; exercise vs. PAHs *p* = 0.002; exercise vs. exercise + PAHs *p* = n.s.; PAHs vs. exercise + PAHs *p* = 0.002) ^A^. (**e**) eNOS *p* = 0.038 (control vs. exercise *p* = 0.037; control vs. PAHs *p* = n.s.; control vs. exercise + PAHs *p* = n.s.; exercise vs. PAHs *p* = n.s.; exercise vs. exercise + PAHs *p* = n.s.; PAHs vs. exercise + PAHs *p* = n.s.) ^A^. (**f**) Ahr *p* = 0.0004 (control vs. exercise *p* = n.s.; control vs. PAHs *p* = 0.030; control vs. exercise + PAHs *p* = n.s.; exercise vs. PAHs *p* = 0.005; exercise vs. exercise + PAHs *p* = n.s.; PAHs vs. exercise + PAHs *p* = 0.0007) ^K-W^. (**g**) RelA *p* = 0.002 (control vs. exercise *p* = n.s.; control vs. PAHs *p* = 0.003; control vs. exercise + PAHs *p* = n.s.; exercise vs. PAHs *p* = 0.041; exercise vs. exercise + PAHs *p* = n.s.; PAHs vs. exercise + PAHs *p* = 0.003) ^A^. (**h**) IL-6 (*p* = 0.582) ^K-W^. (**i**) TNF-α (*p* = 0.849) ^K-W^. Relative quantification was calculated using the reference genes RPL32 and B2m. Data are presented as mean ± SEM. Different letters show statistically significant differences. ^A^ Ordinary one-way ANOVA with Tukey’s multiple comparisons test. ^W-A^ Welch’s ANOVA test with Games-Howell’s multiple comparison test. ^K-W^ Kruskal–Wallis with Dunn’s test multiple comparison analysis. (n = 10 per group).

We found significant differences in gene expression between VCAM-1 (*p* = 0.005), ICAM-1 (*p* = 0.001), E-selectin (*p* = 0.029), Nrf2 (*p* = 0.0001), eNOS (*p* = 0.038), Ahr (*p* = 0.0004), and RelA (*p* = 0.002). No differences were detected regarding IL-6 (*p* = 0.582) and TNF-α (*p* = 0.849) (Figure 4).

### 3.5. Protein Expression

**Figure 5:**
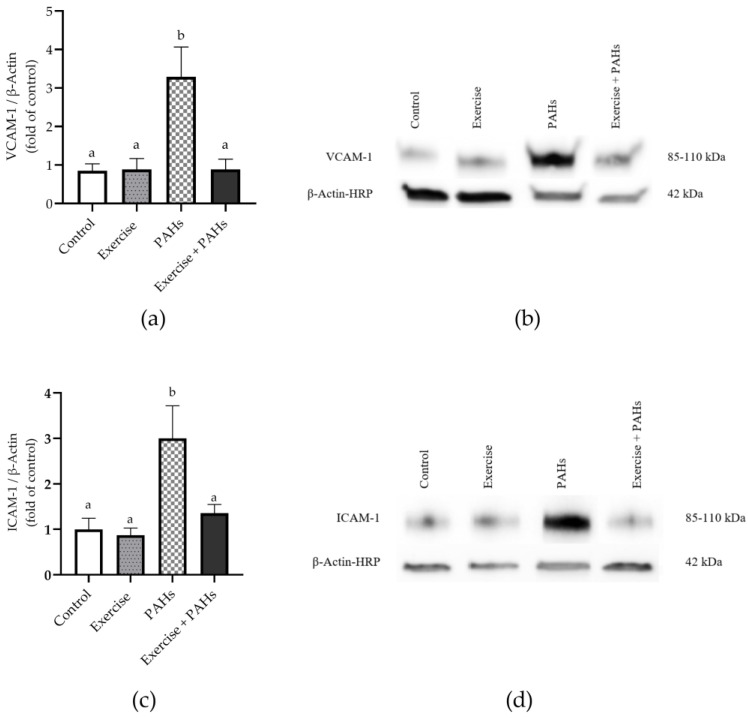
Relative protein expression of adhesion molecules in aortic tissue. (**a**) Relative protein expression of VCAM-1 *p* = 0.006 (control vs. exercise *p* = n.s.; control vs. PAHs *p* = 0.034; control vs. exercise + PAHs *p* = ns, exercise vs. PAHs *p* = 0.020, exercise vs. exercise + PAHs *p* = n.s.; PAHs vs. exercise + PAHs *p* = 0.024) ^K-W^. (**b**) Representative image of VCAM-1 blotting and β-actin loading control. (**c**) Relative protein expression of ICAM-1 *p* = 0.003 (control vs. exercise *p* = n.s.; control vs. PAHs *p* = 0.010; control vs. exercise + PAHs *p* = n.s.; exercise vs. PAHs *p* = 0.007; exercise vs. exercise + PAHs *p* = n.s.; PAHs vs. exercise + PAHs *p* = 0.030) ^A^. (**d**) Representative image of the blotting of ICAM-1 and the β-actin loading control. Data are presented as mean ± SEM. Different letters show statistically significant differences. ^A^ Ordinary one-way ANOVA with Tukey’s multiple comparisons test. ^K-W^ Kruskal–Wallis with Dunn’s test multiple comparison analysis. (n = 10 per group).

The protein expression of VCAM-1 (*p* = 0.006) and ICAM-1 (*p* = 0.003) showed significant differences between the groups, its expression being higher in both cases for the PAH exposure group (Figure 5). 

## 4. Discussion

We recently reported the use of the intranasal route for the administration of a mixture of PAHs derived from previously characterized air pollution [11], which was used in this study with a concentration of 50 µg [27]. The concentrations of PAHs administered in this study are within the ranges published in various studies and are adequately summarized in the review of Møller et al. 2016 [28]. 

The protocol of acclimatization to physical training allowed the animals to adapt to the equipment and the subsequent protocol. The process of adaptation to physical exercise is essential for training responses to be adequate and reliable [29,30,31]. After the intervention, in the maximum exercise capacity test, differences were found between the groups for the variables time, distance, and maximum speed, as well as intragroup differences for the control, exercise, and exercise + PAHs groups in all variables analyzed, but not for the exposure group only to PAHs, where differences were only found for the time variable. In previous studies that enquired about exposure to air pollutants and physical exercise, it has been reported that there was an increase in physical capacity measured through different parameters (speed, distance, or time) in groups subjected to physical exercise, alone or in combination with exposure to pollutants (PM_10_, PM_2.5_ and particles derived from diesel combustion), but this was less frequently observed in the control groups [20,21,22,32].

Once the exercise intervention protocol was developed, statistical differences were found between the groups for the cytokines IL-6, IL-1β, TNF-α, and INF-gamma. On the contrary, IL-17A and IL-10 did not show differences between the groups. For all evaluated cytokines, there was an increase in serum concentrations in the groups exposed to PAHs. Among the deleterious effects described in the literature of exposure to various environmental air pollutants is an increase in pro-inflammatory cytokines evaluated in the blood. In various studies, exposure to PAHs has been found to generate an increase in pro-inflammatory cytokines in the blood, with IL-6, IL-8, and TNF-α being the most studied markers [33,34,35]. Inhaled particles from wood combustion (including PAHs) cause inflammatory responses in the lung; therefore, high particle dose, reactivity of inhaled particles, or failure to remove these particles can cause ‘spillover’ inflammatory responses in the blood that cause systemic inflammation [36]. Clinical studies of wood smoke exposure have consistently found significantly elevated blood markers of inflammation, including IL-1β, IL-6, IL-8, and TNF-α [37]. 

In a study carried out on workers in a coal processing plant, an increase in IL-6 concentration in the blood was observed, showing a direct association with PAHs metabolites measured in urine and demonstrating a dose-dependent relationship in the increase in this inflammatory marker [33]. Another study carried out in young and adult subjects evaluated the variation in various pro-inflammatory cytokines measured in blood in contexts of exposure to PM_10_, PM_2.5–10_, PM_2.5_, PM_1–2.5_, and PM_1_, finding that in older adults, there was an increase in IL-6 for all contaminant sizes, but not in young subjects who had an increase in exposure to all contaminants sizes, except PM_1_ [38]. In an animal model, serum collected from C57BL/6 mice exposed to oak smoke (PM_2.5_) for 24 h was found to be capable of inducing pro-inflammatory responses by increasing the concentration of IL-6 and the expression of adhesion molecules VCAM-1 and ICAM-1 in murine endothelial cells [39]. 

IL-17A is known to activate the induction of IL-6, IL-8, and granulocyte colony stimulating factor (G-CSF) in non-immune cells such as fibroblasts and epithelial cells, in part through the activation of the transcription factor NF-κB. The main gene targets for IL-17 include pro-inflammatory chemokines, hematopoietic cytokines, acute phase response genes, and antimicrobial substances [40]. In a study conducted in mice subjected to a laboratory environment (a clean air environment) and environmental pollution from two underground parking sites (traffic pollution), it was shown that under the two environmental traffic pollution conditions, there was an increase in IL-17A compared to levels in the clean air environment [41].

Physical training can promote innumerable benefits for the body with powerful effects on the cardiovascular system. Among the systemic benefits associated with the execution of physical exercise, the regulation of an optimal REDOX balance and a reduction in inflammation are prominent. Skeletal muscle is an endocrine organ that can produce and release myokines into the bloodstream (particularly during muscle contraction), where they function locally and/or systemically to cause multiple beneficial effects, including decreased inflammation and insulin resistance. The anti-inflammatory environment created by exercise training is believed to be largely mediated by myokines, which also protect arteries against the progression of atherosclerosis and stenosis and preserve the stable phenotype of pre-existing atherosclerotic plaques [42]. In addition, a reduction in the levels of pro-inflammatory mediators IL-18, CRP, TNF-α, and IL-1β, as well as a marked increase in IL-10, has been identified in the blood of exercised individuals [43].

The increase in serum pro-inflammatory cytokines generates deleterious effects in multiple tissues, first affecting the endothelial cells of the vascular system. As an adaptive response to pathological stimuli, the endothelium changes its phenotypic characteristics in a process called ‘endothelial activation’ characterized by an increase in the expression of the adhesion molecules P-selectin, E-selectin, ICAM-1, and VCAM-1. This damages the barrier function of the endothelium, which favors the diapedesis of leukocytes; it increases vascular tone by reducing the production of nitrogen oxides and reduces resistance to thrombosis. [44]. This endothelial activation process has been described as the factor that initiates the formation of atheromatous plaques in vascular tissue. Nitric oxide (NO), produced by the enzyme eNOS in endothelial tissue, is a gasotransmitter generated by the ‘healthy’ endothelium that has effects on vascular tone and prevents smooth muscle cell proliferation and migration, leukocyte adhesion, and platelet aggregation. Adequate eNOS activity protects against pathological vascular remodeling, hypertension, atherosclerosis, and complications associated with diabetes [45].

In this protocol, statistically significant differences were observed in the gene expression of ICAM-1, VCAM-1, E-selectin, and eNOS. Furthermore, significant differences in the gene expression of Nrf2, Ahr, and RelA were identified following the established protocol. The existing literature has established a direct correlation between Nrf2, Keap1, and the response to environmental stress [46,47]. This regulatory system is modulated through interactions between Nrf2 and the cytosolic repressor protein Keap1. Upon dissociation of this complex, Nrf2 is released and translocates to the nucleus, stimulating transcription. Nrf2 target genes encompass enzymes related to glutathione synthesis and conjugation, antioxidant enzymes, drug-metabolizing enzymes, transporters, and enzymes of the pentose phosphate pathway [48,49]. 

In this sense, it is known that E-selectin and P-selectin are responsible for the ‘rolling’ of circulating cells on endothelial surfaces, which facilitates their anchoring to the endothelium [50]. In vitro studies of bronchial epithelial cells exposed to pollutants derived from diesel combustion found no differences in E-selectin gene expression [51]. On the other hand, in human brain microvascular endothelial cells exposed to tobacco smoke, an increase in the expression of P-selectin, VCAM-1, and E-selectin has been reported compared to controls [52]. In addition, in HUVEC cells exposed to PM_2.5_ and PM_10_, an increase in the expression of early (E-selectin and P-selectin) and late (ICAM-1, PECAM-1, VCAM-1) adhesion molecules has been reported [53]. For its part, TNF-α acts on vascular endothelial cells, enhancing the expression of adhesion molecules, which results in inflammatory cell adhesion between endothelial cells and leukocytes, infiltration, and neutrophil degranulation [54]. In vitro experiments have shown that the stimulation of endothelial cells of the human coronary artery with TNF-α increased the expression of ICAM-1. In animal models exposed to PM_2.5_, eNOS phosphorylation was observed to decrease, leading to a decrease in nitrogen oxide production, which promotes endothelial dysfunction [55]. In addition, a decrease in the production of nitric oxide has been observed in RAW 246.7 cells exposed to PM_2.5_ sample containing PAHs, showing a negative correlation between the production of nitric oxide and the concentration of PAHs [56]. 

The protein expression of ICAM-1 and VCAM-1 showed significant differences, increasing in the exposure group, and decreasing in expression in the groups that performed physical exercise. In this sense, it is known that atherosclerosis is influenced by interactions between cell adhesion molecules, where the expression of these molecules on the cell surface in response to pathophysiological stimuli interferes with the interaction between the endothelium and blood cells and is essential for the development of atherosclerosis [57]. ICAM-1 and VCAM-1 are closely related in structure and function. Both are members of the cytokine-inducible Ig gene superfamily that binds to leukocyte integrins [58]. When endothelial cells are activated, they express monocyte chemoattractant proteins-1, IL-8, ICAM-1, VCAM-1, E-selectin, P-selectin, and other inflammatory factors that attract lymphocytes and monocytes bound to endothelial cells and infiltrate the arterial wall [59]. Regarding this, it has been observed that in mice exposed to PM_2.5_ by intratracheal instillation, there is an increase in VCAM-1 expression along with an increase in neutrophil infiltration of alveolar tissue, which favors adhesion, chemotaxis, and migration to the tissue [60]. Furthermore, PM_2.5_ has been shown to induce oxidative stress, which generates an increase in the expression of ICAM-1 and VCAM-1 by activating the ERK/AKT/NF-κB signaling pathway, which promotes the adhesion of monocytes to endothelial cells [61]. In a study carried out in older adults exposed to air pollution, it was determined that there were higher levels of VCAM-1 after days of exposure to black carbon and higher levels of ICAM-1 after weeks of exposure. Furthermore, ICAM-1 and VCAM-1 were also found to be higher after short- and medium-term exposure to PM_2.5_ and sulphates [62]. Incorporating the analysis of protein expression of E-selectin, P-selectin, and PECAM-1 in aortic tissue in future studies can improve our understanding of the increased expression of adhesion molecules and endothelial permeability.

As previously cited, there is no consensus in the literature on the practice of physical exercise in environments with air pollution. Therefore, increased levels of air pollution have been documented to be related to a higher incidence of asthma and chronic obstructive pulmonary disease (COPD), a higher incidence of and mortality from lung cancer, reduced lung function, and a deficit in lung development during childhood [63]. The characteristic features of chronic obstructive pulmonary disease (COPD) include inflammation and remodeling of the lower airways and lung parenchyma, together with the activation of inflammatory and immune processes. Due to the growing habit of cigarette smoking around the world, the prevalence of COPD is increasing globally. Furthermore, in a review of the literature related to air pollution and exercise in humans, it was reported that under conditions of air pollution (especially PM) there was a reduction in physical performance, as measured by standardized physical tests; however, the authors did not elaborate on the long-term effects of physical exercise programs [64]. Similarly, a study in healthy young men found that short-term exposure to elevated ambient levels of PM_2.5_ and SO_2_ during submaximal exercise was associated with a decrease in forced expiratory volume in 1 s (FEV1) and forced vital capacity (FVC) in a model adjusted for BMI, allergic status, and pattern of physical activity [65]. In contrast, in a study carried out with 122 adults, the inhalation of PM caused a decrease in lung function, while physical activity acted as an acute bronchodilator that could provide a protective effect against deleterious effects on lung function related to exposure to PM, at least in the short term [66].

However, there is evidence that in the long term, the beneficial effects of physical exercise on cardiovascular health are more relevant than the harmful effects caused by air pollution [18,19]. Thus, in animal models, a decrease in lung inflammation has been found, mediated by a better antioxidant response in mice subjected to high-intensity aerobic exercise together with exposure to pollutants derived from diesel exhaust particles, PM_2.5_ and PM_10_ [20,21,22]. In turn, in a systematic review of the literature that evaluated the impact on human health of active transport (walking or cycling), it was determined that the health benefits are greater with this type of transport even in contexts of air pollution (especially PM_2.5_); however, the study recognizes that estimating the risk to health is complex given the geographical and particular characteristics of the composition of this PM_2.5_ [67]. Similarly, another review concluded that active transport has substantial overall health benefits that far outweigh the negative effects of exposure to air pollution in all but the most extreme air pollution [68]. In this same line, a study evaluating the response of blood pressure to exposure to black carbon found no risk attributable to exposure to this pollutant with respect to blood pressure but determined that subjects who performed moderate- to high-intensity physical activity registered a significant decrease in blood pressure, highlighting that follow-up was only 7 days [69].

Another study enquired about the risk of physical exercise and hospitalizations for asthma and COPD, determining that the beneficial effects of physical activity and the adverse effects of long-term exposure to air pollution associated with vehicular traffic (NO_2_) were independent factors in hospitalizations for asthma and COPD. Moreover, the benefits of physical activity were not reduced for those living in areas with high air pollution. Therefore, they concluded that the long-term benefits of physical activity in preventing the development of asthma and COPD in healthy elderly subjects can outweigh the risks associated with increased exposure to air pollution, including during physical activity, in addition to potentially allowing effective control of the disease in patients with COPD [70]. In this same sense, in a study that investigated the effects of physical exercise in the context of exposure to PM_2.5_ for men with and without allergic conditions, it was concluded that exposure to air pollution during short-term and moderate-intensity exercise did not have an acute negative impact on respiratory and cardiovascular function in healthy young men [71].

Furthermore, in a longitudinal study carried out in Taiwan that included 384,130 adults and investigated deaths from natural causes, physical exercise, and exposure to PM_2.5_, it was concluded that moderate-intensity exercise and high-intensity exercise reduce the risk of death from natural causes, regardless of exposure levels. Furthermore, the results suggest that physical exercise is a safe strategy for improving health, even for people residing in relatively polluted regions [72], showing that regular physical activity is associated with lower markers of systemic inflammation at different levels of air pollution [73]. In a 20-year longitudinal study that included 104,990 women, it was determined that physical activity had a protective effect against cardiovascular disease, acute myocardial infarction, stroke, and overall mortality at all levels of long-term exposure to PM_2.5_ [74].

Although the characterization of exposure to a mixture of PAHs yields intriguing information about their potential roles in inflammation and cardiovascular conditions, this study is subject to some limitations. The small number of animals per group, derived from the lack of previous data on the administered PAH model, poses a limitation in the generalization of the results. Furthermore, the five-week duration of exposure, while revealing significant effects, may not fully capture the widespread cardiovascular impacts observed in individuals exposed to chronic air pollution.

The study encountered a significant limitation regarding the ability to conduct thorough biochemical analyses of blood, encompassing key parameters such as glucose, bicarbonate, and oxygen levels. This constraint stemmed from the restricted sample volume per animal, which allowed an analysis focused exclusively on serum cytokines. Additionally, we were unable to evaluate PAH metabolites, such as 1-hydroxypyrene and 1-OH-pyrene, which are typically evaluated in urine. Incorporating such analyzes could strengthen the mechanistic ideas of the study. The exercise protocol could be improved by adapting the workload based on the animal’s performance, which would require an unplanned intermediate stress test. Furthermore, the inclusion of various exercise modalities, including high-intensity interval training (HIIT), known for its cardiovascular benefits and improved endothelial function, would have enriched the study’s exercise-related observations.

## 5. Conclusions

This investigation used a protocol for the administration of PAHs present in air pollution through nasal instillation [27] and demonstrated that it is a simple method that allows the incorporation of these compounds into the respiratory system without associated damage and can be used in animal models of physical exercise. 

Administration of a dose of a mixture of PAHs present in air pollution for 5 weeks yielded an increase in circulating inflammatory cytokines, demonstrating a state of systemic inflammation characteristic of cardiovascular diseases. Furthermore, it was possible to determine that exposure to PAHs generated alterations in the aortic tissue, increasing the expression of gene markers of endothelial dysfunction, REDOX imbalance, and, to a lesser degree, inflammatory markers. Similarly, there was an increase in the protein expression of markers of endothelial dysfunction in the same tissue. These changes observed in the aortic tissue can be positively modulated by aerobic physical exercise, and it could be projected that intervention with physical exercise in the context of exposure to PAHs present in air pollution decreases the risk of cardiovascular disease related to these markers, which requires future research.

## Figures and Tables

**Table 1:** The primer sequences used for the PCR analysis.

Gene	Accession Number	Sequence Forward	Sequence Reverse	Fragment Length (bp)
ICAM-1	NM_010493	TTCTCATGCCGCACAGAACT	TCCTGGCCTCGGAGACATTA	73
VCAM-1	NM_011693	CTGGGAAGCTGGAACGAAGT	GCCAAACACTTGACCGTGAC	115
E-selectin	NM_011345	AGCCTGCCATGTGGTTGAAT	CTTTGCATGATGGCGTCTCG	197
eNOS	NM_008713	GCTCCCAACTGGACCATCTC	TCTTGCACGTAGGTCTTGGG	121
Nrf2	NM_010902	GATGACCATGAGTCGCTTGC	CCTGATGAGGGGCAGTGAAG	73
Ahr	NM_013464	TAAAGTCCACCCCTGCTGAC	CATTCAGCGCCTGTAACAAGA	108
RelA	NM_009045	CCTGGAGCAAGCCATTAGC	CGCACTGCATTCAAGTCATAG	99
IL-6	NM_031168	CCCCAATTTCCAATGCTCTCC	CGCACTAGGTTTGCCGAGTA	141
TNF-α	NM_013693	ATGGCCTCCCTCTCATCAGT	TTTGCTACGACGTGGGCTAC	97
RPL32	NM_172086	TAAGCGAAACTGGCGGAAAC	CATCAGGATCTGGCCCTTGA	73
B2m	NM_009735	ACTGACCGGCCTGTATGCTA	CAATGTGAGGCGGGTGGAA	125

ICAM-1 (intercellular adhesion molecule 1); VCAM-1 (vascular cell adhesion molecule 1); E-selectin (selectin, endothelial cell); eNOS (nitric oxide synthase endothelial cell); Nrf2 (nuclear factor erythroid-derived 2-like 2); Ahr (aryl hydrocarbon receptor); RelA (transcription factor p65); IL-6 (interleukin 6); TNF-α (tumor necrosis factor α); RPL32 (ribosomal protein L32); B2m (beta-2 microglobulin).

## Data Availability

All data are described in the manuscript.
